# SMURF1-mediated ubiquitination of ARHGAP26 promotes ovarian cancer cell invasion and migration

**DOI:** 10.1038/s12276-019-0236-0

**Published:** 2019-04-19

**Authors:** Xuri Chen, Shaoyun Chen, Yao Li, Yanling Gao, Shuying Huang, Hongping Li, Yuanfang Zhu

**Affiliations:** 1 Department of Obstetrics and Gynecology, Bao’an Maternity and Child Health Hospital, Jinan University, Shenzhen, 518100 China; 2 Maternal-Fetal Medicine Institute, Bao’an Maternity and Child Health Hospital, Jinan University, Shenzhen, 518100 China; 3 Shenzhen children’s Hospital, Shenzhen, 518000 China

**Keywords:** Targeted therapies, Ubiquitylation

## Abstract

Rho GTPase-activating protein 26 (ARHGAP26) is a negative regulator of the Rho family that converts the small GTP-binding protein RhoA (GTP-RhoA) to its inactive GDP-bound form and is a putative tumor suppressor gene associated with cell growth and migration. Here, the involvement of ARHGAP26 in ovarian cancer cell proliferation and migration was investigated. In this study, low ARHGAP26 expression was observed in ovarian cancer tissues and was associated with a poor overall survival and higher β-catenin expression in patients with ovarian cancer. A2780 and HEY cells with ARHGAP26 upregulation showed decreased cell proliferation, migration, and invasion, along with decreased GTP-RhoA, β-catenin, VEGF, MMP2, and MMP7 expression. ARHGAP26 upregulation in A2780 cells also inhibited lung metastasis in vivo. SKOV3 cells with ARHGAP26 downregulation demonstrated an inverse effect, which was inhibited by ARHGAP26 overexpression or DKK1, an antagonist of the β-catenin pathway. SMURF1, an E3 ubiquitin ligase, interacted with and induced ubiquitination of ARHGAP26. ARHGAP26 upregulation in SKOV3 cells significantly inhibited SMURF1 upregulation-induced cell migration and invasion. Overall, SMURF1-mediated ubiquitination of ARHGAP26 may promote invasion and migration of ovarian cancer cells via the β-catenin pathway.

## Ovarian cancer: Suppressing the spread

A member of a protein family known to suppress tumors could help tackle ovarian cancer metastasis. Ovarian cancer remains challenging to treat because the molecular mechanisms inherent in the disease are poorly understood. Hongping Li at Shenzhen Children’s Hospital, Yuanfang Zhu at Bao’an Maternity and Child Health Hospital, and co-workers showed that low levels of a tumor-suppressing protein, Rho GTPase-activating protein 26 (ARHGAP26), promote the invasion and migration of ovarian cancer cells. The team took tumor and healthy tissue samples from 85 patients and confirmed that reduced levels of ARHGAP26 in the cancerous tissues coincided with an increase in an enzyme involved in protein degradation. This enzyme-led destruction of ARHGAP26 may enable cancer metastasis. The team backed up their hypothesis by observing reduced cancer cell proliferation in mice with high levels of ARHGAP26.

## Introduction

Ovarian cancer is one of the three most common malignant tumors of female reproductive organs and the predominant pathological type is epithelial carcinoma, accounting for 85–90% of malignant ovarian tumors^1^. After cervical and endometrial cancer, the incidence of ovarian cancer ranks third, but the mortality rate is the highest compared with other malignant tumors of female reproductive organs^2^. More than 200,000 new cases are diagnosed and 100,000 people die each year^3^. The 5-year overall survival rate of ovarian cancer patients is only 47% due to the lack of early clinical symptoms, practical early diagnosis, and lasting and effective treatment, as well as the high susceptibility to chemotherapy resistance and relapse^4,5^. Therefore, a better understanding of the potential pathogenic genes is urgently required for improved ovarian cancer treatment.

Rho GTPase-activating protein 26 (ARHGAP26) belongs to the small G protein family that can hydrolyze active Rho GTPase into inactive Rho GDP and negatively regulates RhoA^6^. RhoA belongs to the Rho GTPase superfamily and is abnormally expressed in many malignant tumors and involved in the tumor invasion and metastasis^7,8^, contributing to the occurrence and development of cancer. *ARHGAP26* is a recognized tumor suppressor gene that was found inactivated in acute myeloid leukemia and an independent prognostic factor for acute myeloid leukemia^9^. Deletion and mutation of ARHGAP26 can lead to promyelocytic leukemia^10^, suggesting tumor suppressive activity of ARHGAP26. ARHGAP26 was downregulated in glioblastoma and associated with cell proliferation and migration^11^. Emerging evidence has linked other Rho GAPs to the development and progression of ovarian cancer^12^. However, the molecule mechanism and regulation of ARHGAP26 in ovarian cancer tumorigenesis is still unclear.

Ubiquitination is a posttranslational modification in which ubiquitin is attached to one or more lysine residues of cellular proteins through a series of enzymatic cascade reactions^13^. Similar to phosphorylation, ubiquitination alters the stability, conformation, or localization of the target proteins through reversible covalent modification, thereby regulating signal transduction, protein–protein interactions, gene transcription, and other biological processes^14^. Ubiquitination is catalyzed by a ubiquitin-activating enzyme E1, ubiquitin-conjugating enzyme E2, and ubiquitin ligase enzyme E3, the latter of which regulates the specificity of substrates in the ubiquitin proteasomal system. Smad ubiquitination regulatory factor 1 (SMURF1) is an E3 ubiquitin–protein ligase and increased SMURF1 expression has been observed in patients with ovarian cancer^15^, promotes RhoA ubiquitination, and regulates cell growth and metastasis^16^. Nevertheless, the cellular function of SMURF1 and its role in regulation of ARHGAP26 in ovarian cancer remain largely unknown.

In this study, we report that ARHGAP26 is downregulated, whereas β-catenin and SMURF1 are upregulated in ovarian cancer patients. ARHGAP26 upregulation inhibited ovarian cancer cell proliferation, invasion, and migration in vitro and lung metastasis in vivo. ARHGAP26 downregulation promoted ovarian cancer cell invasion and migration by activating the β-catenin pathway. SMURF1 upregulation promoted ubiquitination of ARHGAP26 and induced ovarian cancer cell migration and invasion, which were inhibited by ARHGAP26 upregulation. These data suggest that SMURF1-mediated ubiquitination of ARHGAP26 may promote ovarian cancer cell invasion and migration via the β-catenin pathway.

## Materials and methods

### Bioinformatics

Gene expression data were obtained from The Cancer Genome Atlas (TCGA, https://tcga-data.nci.nih.gov/tcga/) for ovarian cancer projects, including 568 cases with tumor tissues and 8 cases with adjacent noncancerous tissues. Gene-set enrichment analysis (GSEA) was used to identify the pathways that were significantly enriched between patients with high and low ARHGAP26 expression.

### Tissue samples

In total, 85 cases of tumor tissues and their corresponding adjacent noncancerous tissues were obtained from ovarian cancer patients in Bao’an Maternity and Child Health Hospital recruited from October 2012 to March 2017. Human ovarian cancer and adjacent normal tissues were immediately snap-frozen in liquid nitrogen and stored at −80 °C until immunohistochemistry (IHC) was performed^17^. All of the patients provided signed informed consent. The medical ethics committee of Bao’an Maternity and Child Health Hospital approved the retrieval method for cancer specimens.

### Cell culture and transfection

The human ovarian cancer cell lines OVCAR3, SKOV3, A2780, HEY, and CAOV3, and nonmalignant human ovarian surface epithelial cells IOSE80 were all purchased from the Shanghai Institute of Biochemistry and Cell Biology (Shanghai, China), and cultured in an incubator with 95% humidity and 5% CO_2_ at 37 °C in RPMI-1640 medium (HyClone, Logan, UT, USA) with 10% fetal bovine serum (Gibco Lab, Grand Island, NY, USA) and 1.0% penicillin–streptomycin solution (Solarbio, Beijing, China).

A2780 and HEY cells were cultured in six-well plates at 2 × 10^5^ cells/well overnight and transduced with a lentiviral vector encoding ARHGAP26 (pLVX-Puro-ARHGAP26) or with a blank pLVX-Puro lentivirus as the negative control (blank vector). To silence ARHGAP26 expression, SKOV3 cells under the same culture conditions were transfected with ARHGAP26 small interfering RNA (siRNA) (siRNA-1, position 439–457, 5′-GCUGGACAAGACCAACAAA-3′; siRNA-2, position 1140–1158, 5′-CCAUCAGUCCCUACACCAU-3′; siRNA-3, position 1213–1231, 5′-GCACUACUGUACAUAUCAA-3′) or a control siRNA (siNC) using Lipofectamine 2000 (Invitrogen, Carlsbad, CA, USA) according to the manufacturer’s instructions and incubated for 6 h at 37 °C. After being incubated in complete RPMI-1640 medium for 48 h, SKOV3 cells were treated with or without 200 ng/mL DKK1. Otherwise, SKOV3 cells were transduced with pLVX-Puro-WWP1, pLVX-Puro-CBL, pLVX-Puro-NEDD4, pLVX-Puro-MDM2, pLVX-Puro-SMURF1, or pLVX-Puro-ARHGAP26 alone or in combination with one another.

### CCK-8 assay

A Cell Counting Kit (CCK-8) assay was used to measure cell proliferation. Cells were cultured in 96-well plates at 3 × 10^3^ cells/well for 24, 48, and 72 h. Then, each well was filled with 10 μL of CCK-8 solution and incubation was continued at 37 °C for an additional 1 h. The absorbance of each well was determined at 450 nm.

### Transwell assay

Cells were serum-starved in serum-free RPMI-1640 medium for 24 h and cell suspensions (9 × 10^4^/well) were plated in Transwell chambers with (invasion assay) or without (migration assay) Matrigel coating. Then, 700 μL of RPMI-1640 medium supplemented with 10% fetal bovine serum was added to the lower chamber. After 48 h of incubation, the cells were fixed, stained, photographed, and counted under a light microscope (Olympus Corporation, Tokyo, Japan) at ×200 magnification.

### Quantitative real-time PCR

Total RNA was extracted from ovarian cancer tissues and cell lines using Trizol reagent (Invitrogen) according to the manufacturer’s protocol and reverse-transcribed with a RevertAid First Strand cDNA Synthesis Kit (Thermo Fisher, Waltham, MA, USA). Quantitative real-time PCR was carried out using SYBR green PCR Master Mix (Fermentas, Vilnius, Lithuania) on an ABI7500 system following the manufacturer’s instructions. The forward and reverse primers used for quantitative real-time PCR amplification were as follows: ARHGAP26 (5′-AATTCCAGCAGCAGCTTAC-3′ and 5′-TTCAGCTTTGCAGGCATAC-3′); SMURF1 (5′-CTGGCAAGCGGTGGAGAC-3′ and 5′-CCGGTTAAAGCAGGTATGGG-3′); CBL (5′-CATCTGCCAATGCCATTTATTC-3′ and 5′-GCTATCAATCTGCTGGTCGC-3′); NEDD4 (5′-AGTTTGTCACTGGCACATCTCG-3′ and 5′-CAGCTTTTCAGGAGTACCCCAC-3′); MDM2 (5′-GGGCTTTGATGTTCCTGATTG-3′ and 5′-TTCTTTGTCTTGGGTTTCTTCC-3′); WWP1 (5′-ATAATGCGTCTGTCACGGGTAC-3′ and 5′-GCTGTCTTGATTTGGCTGCTTC-3′); GAPDH (5′-AATCCCATCACCATCTTC-3′ and 5′-AGGCTGTTGTCATACTTC-3′). The relative abundance of genes was quantified using the comparative 2^−ΔΔCt^ method with GAPDH as the internal control.

### Western blotting

Cell lysates were prepared as previously described^17^. An equal amount of protein was incubated with glutathione *S*-transferase-rhotekin (Upstate Biotechnologies, Inc., Lake Placid, NY, USA) for 45 min at 4 °C to collect the active form of RhoA (GTP-RhoA). Immunoblotting was performed with antibodies against ARHGAP26 (Abcam, Cambridge, MA, USA), RhoA (Santa Cruz Biotechnology, Santa Cruz, CA, USA), MMP2 (Abcam), MMP7 (Abcam), SMURF1 (Abcam), CBL (Abcam), NEDD4 (Abcam), MDM2 (Abcam), WWP1 (Abcam), VEGF (Affinity, Cincinnati, OH, USA), β-catenin (Cell Signaling Technology, Danvers, MA, USA), GAPDH (Cell Signaling Technology), and horseradish peroxidase-labeled goat anti-rabbit IgG (Beyotime Biotechnology, Shanghai, China). Proteins on membranes were detected using an ECL system (Amersham Biosciences, Piscataway, NJ, USA).

### Co-immunoprecipitation

Whole-cell lysates obtained by centrifugation were incubated with 2 μg anti-ARHGAP26, anti-SMURF1 or normal IgG antibody, and protein G-Agarose beads (Roche Diagnostics Ltd, Shanghai, China) at 4 °C overnight. The immunocomplexes were then separated via sodium dodecyl-sulfate polyacrylamide gel electrophoresis (SDS–PAGE), and blottings were probed with the indicated antibody.

### Ubiquitination assay

SKOV3 cells were transduced with pLVX-Puro-SMURF1 or blank lentivirus (blank vector). After 48 h, cell lysates were subjected to immunoprecipitation with anti-ARHGAP26 or normal IgG antibody for 4 h at 4 °C. After incubation with protein G-agarose beads at 4 °C for 1 h, the immunocomplexes were separated via SDS–PAGE and subjected to western blotting analysis with anti-ubiquitin antibody.

### Animal experiments

A total of 1 × 10^6^ A2780 or HEY cells transduced with pLVX-Puro-ARHGAP26 or blank vector were collected, resuspended in 100 μL phosphate-buffered saline, and then intravenously injected through the tail vein into 5–6-week-old female BALB/c nude mice (weight: 16–18 g; 6 per group). Mice were killed 21 days after injection and the lung tissues were removed from the xenograft mice and stained with hematoxylin and eosin as previously described^18^. Animal experiments were approved by the Bao’an Maternity and Child Health Hospital institutional ethical committee and performed according to the legal requirements.

### Statistical analysis

Each experiment was performed in triplicate. The data were analyzed using GraphPad Prism 5.0 software (GraphPad Software, Inc., La Jolla, CA, USA) and are presented as the mean ± SD. Differences between groups were calculated using one-way or two-way analysis of variance. All statistical tests were two-sided with an *α*-level of 0.05.

## Results

### Decreased ARHGAP26 expression in ovarian cancer tissues

**Fig. 1:**
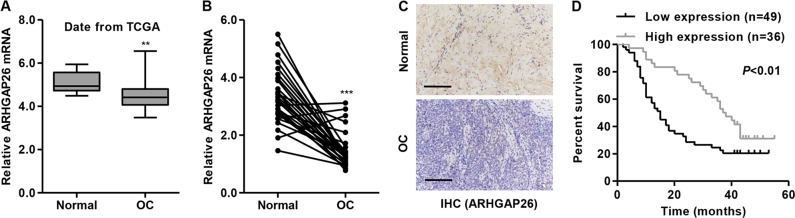
ARHGAP26 expression was decreased in ovarian cancer tissues. ARHGAP26 mRNA expression in ovarian cancer tissues from TCGA (**a**) and our independent hospital (**b**) cohorts was detected. ARHGAP26 protein expression in our independent hospital (**c**) cohorts was detected via immunohistochemistry (IHC). Scale bars: 100 μm. **d** Overall survival of 85 patients with ovarian cancer from the hospital cohort was detected. **P < 0.01 and ***P < 0.001 compared with normal tissues. OC, ovarian cancer; normal, adjacent noncancerous tissues

We analyzed the expression levels of ARHGAP26 in ovarian cancer tissues using TCGA and our independent hospital databases. The results demonstrated downregulated ARHGAP26 mRNA expression in ovarian cancer tissues compared with adjacent noncancerous ovarian tissues from TCGA (Fig. 1a) and independent hospital cohorts (Fig. 1b).

We also examined ARHGAP26 protein expression in ovarian cancer tissues via IHC analysis, which showed that ARHGAP26 protein expression was markedly decreased in tumor tissues; 57.6% (49/85) of ovarian cancer tissues displayed negative or low expression of ARHGAP26 (<25% of tumor cells were positively stained) (Fig. 1c). The correlation between ARHGAP26 expression and the prognosis of patients with ovarian cancer was analyzed with a log-rank (Mantel–Cox) test. The results indicated that the overall survival of patients with lower ARHGAP26 expression was significantly shorter than that of patients with higher ARHGAP26 expression (Fig. 1d), suggesting that decreased ARHGAP26 expression may contribute to poor survival.

### ARHGAP26 overexpression inhibits ovarian cancer cell proliferation

**Fig. 2:**
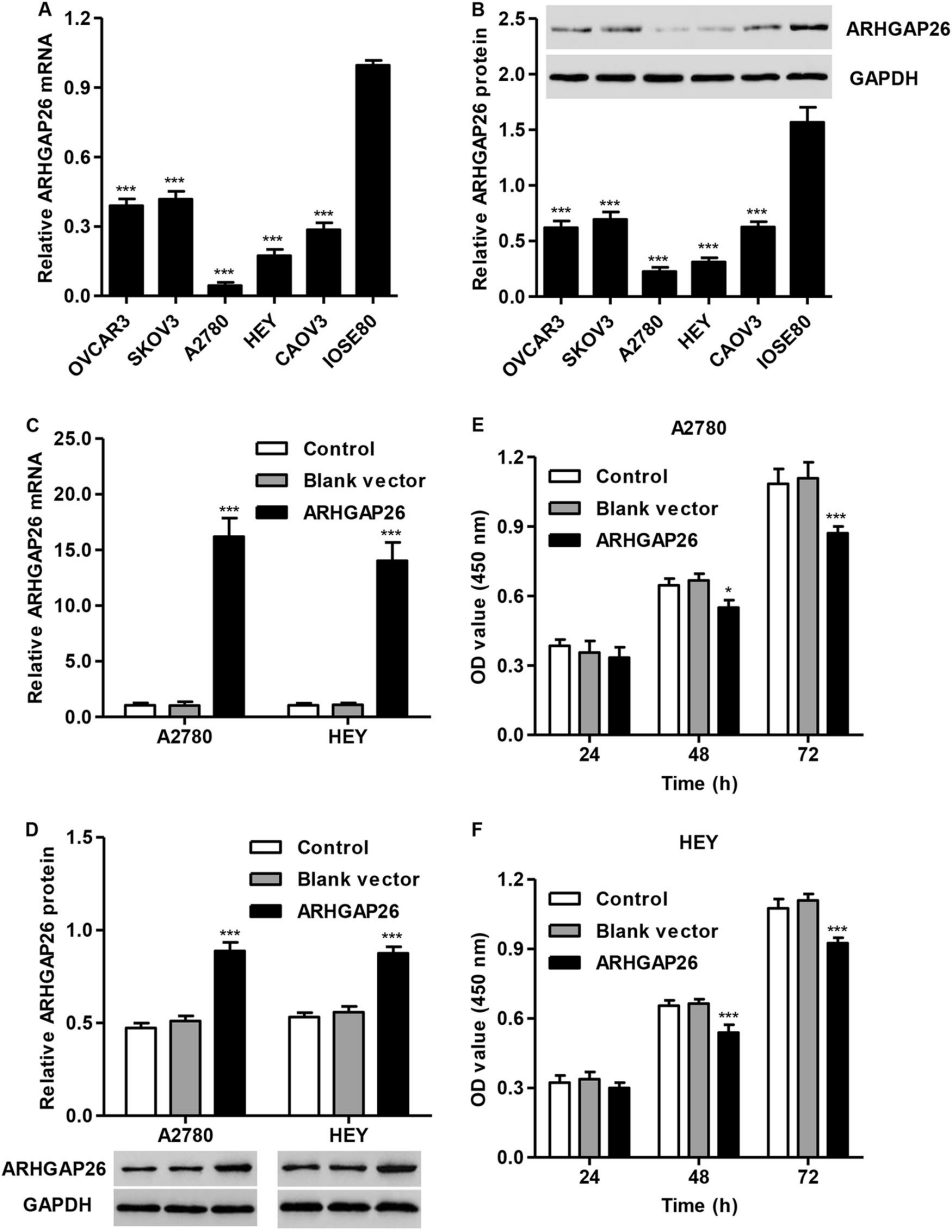
ARHGAP26 upregulation inhibited the proliferation of A2780 and HEY cells. **a**, **b** ARHGAP26 expression in five ovarian cancer cell lines and the nonmalignant human ovarian surface epithelial cell line IOSE80 was detected. A2780 and HEY cells were transduced with ARHGAP26 expression lentivirus or control lentivirus (blank vector). ARHGAP26 expression (**c**, **d**) and cell proliferation (**e**, **f**) was detected via real-time PCR, western blotting, and CCK-8 assays. *P < 0.05 and ***P < 0.001 compared with IOSE80 or control cells

To further examine the role of ARHGAP26 in ovarian cancer tumorigenesis in vitro, ARHGAP26 expression in five ovarian cancer cell lines (OVCAR3, SKOV3, A2780, HEY, and CAOV3) was assessed. As shown in Fig. 2a, b, decreased ARHGAP26 expression was observed in ovarian cancer cell lines compared with that in IOSE80 cells, with the two lowest expression levels detected in A2780 and HEY cells, and the highest expression detected in SKOV3 cells. Therefore, we stably overexpressed ARHGAP26 in A2780 and HEY cells, and its expression in cells transduced with ARHGAP26 overexpression lentivirus was significantly elevated compared with control cells or cells transduced with blank vector (Fig. 2c, d). Moreover, CCK-8 assays demonstrated that the proliferation of A2780 and HEY cells overexpressing ARHGAP26 was significantly suppressed at 48 and 72 h compared with control cells or cells transduced with blank vector (Fig. 2e, f).

### ARHGAP26 overexpression inhibits ovarian cancer cell invasion and migration

**Fig. 3:**
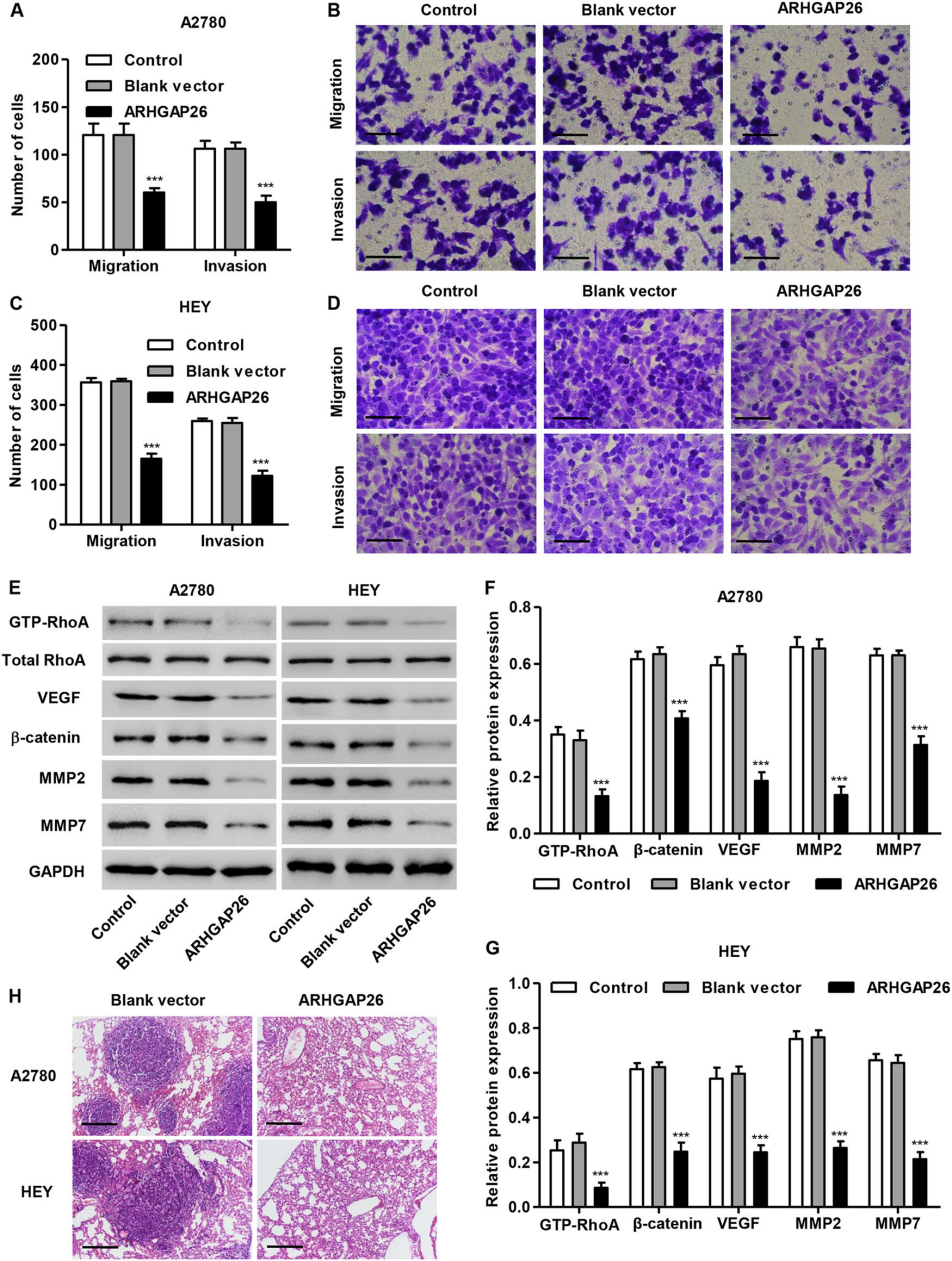
ARHGAP26 upregulation inhibited the ovarian cancer cell invasion and migration. A2780 and HEY cells were transduced with recombined ARHGAP26 expression lentivirus or control lentivirus (blank vector). Cell migration and invasion of A2780 (**a**, **b**) and HEY cells (**c**, **d**) were detected via Transwell analysis, and the expression of GTP-RhoA, total RhoA, β-catenin, VEGF, MMP2, and MMP7 in A2780 and HEY cells was detected via western blotting (**e**–**g**). Scale bars: 100 μm. **h** Histology of lungs metastases from mice intravenously injected with A2780 or HEY cells stably expressing ARHGAP26 or blank vector. Scale bars: 200 μm. ***P < 0.001 compared with control

To determine whether ARHGAP26 affects ovarian cancer cell migration and invasion, Transwell assays were performed. Our data showed that the migration and invasion of A2780 cells overexpressing ARHGAP26 were significantly suppressed by 51.4% and 52.6% compared with control cells (Fig. 3a, b). Similarly, the migration and invasion of HEY cells overexpressing ARHGAP26 were significantly suppressed by 52.3% and 53.1% compared with control cells (Fig. 3c, d). In view of the GSEA data showing that high ARHGAP26 expression is negatively correlated with Wnt/β-catenin signaling (Supplementary Fig. S1), the expression of β-catenin and the downstream effectors VEGF, MMP2, and MMP7, as well as total RhoA and GTP-RhoA, were examined via western blotting. Our results showed that ARHGAP26 overexpression in A2780 and HEY cells significantly inhibited the expression of these proteins, except RhoA, compared with control cells or cells transduced with blank vector (Fig. 3e, g).

Next, we investigated the effect of ARHGAP26 on tumor metastasis in vivo. Tumor metastasis was examined after A2780 or HEY cells stably expressing ARHGAP26 or blank vector were intravenously injected into the tail vein of nude mice. Compared with the mice injected with A2780 or HEY cells transduced with blank vector, less cell metastasis was observed in the lung tissues of nude mice 21 days after injection with A2780 or HEY cells stably expressing ARHGAP26 (Fig. 3h).

### Silencing of ARHGAP26 promotes ovarian cancer cell proliferation, migration, and invasion

**Fig. 4:**
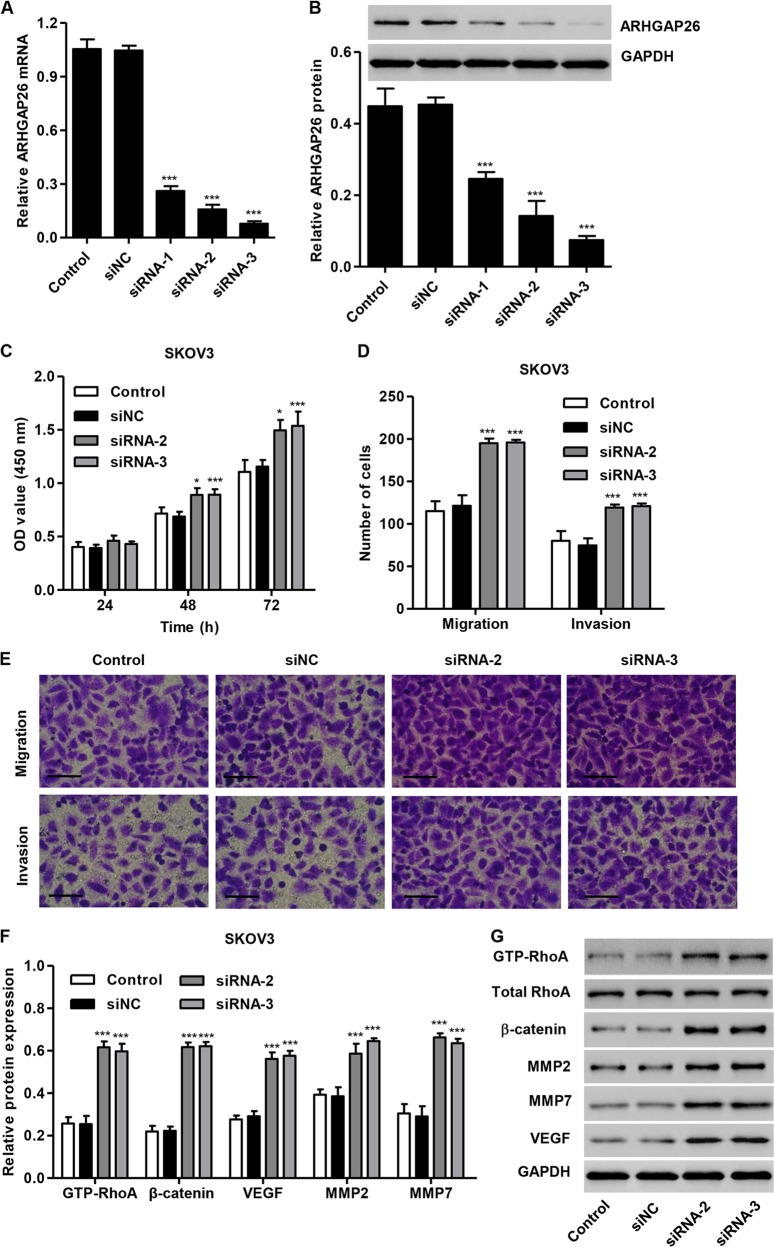
ARHGAP26 downregulation increased the proliferation, invasion, and migration of SKOV3 cells. **a**, **b** SKOV3 cells were transfected with three siRNAs targeting AGRHAP26 or with control siRNA (siNC), and AGRHAP26 expression was detected via real-time PCR and western blotting. SKOV3 cells were transfected with siRNA-2 and siRNA-3. SKOV3 cell proliferation (**c**), migration, and invasion (**d**, **e**) were detected via CCK-8 and Transwell assays, and the expression of GTP-RhoA, total RhoA, β-catenin, VEGF, MMP2, and MMP7 (**f**, **g**) was detected via western blotting. Scale bars: 100 μm. *P < 0.05 and ***P < 0.001 compared with control

To further explore the role of ARHGAP26 in ovarian tumorigenesis, ARHGAP26 was silenced using siRNA in SKOV3 cells. ARHGAP26 expression in SKOV3 cells transfected with siRNA-1, siRNA-2, or siRNA-3 was significantly decreased by 75.6%, 87.1%, and 92.6% at the mRNA level and by 45.3%, 69.7%, and 83.5% at the protein level compared with control cells, respectively (Fig. 4a, b). Therefore, siRNA-2 and siRNA-3 were used in our following experiments. SKOV3 cells transfected with siRNA-2 or siRNA-3 showed increased cell proliferation, migration, and invasion compared with control cells or cells transfected with siNC (Fig. 4c, e). In addition, silencing of ARHGAP26 significantly increased the expression level of GTP-RhoA, β-catenin, VEGF, MMP2, and MMP7 in SKOV3 cells (Fig. 4f, g). These data indicated that ARHGAP26 may be a potential regulator of tumor migration and invasion in ovarian cancer.

### DKK1 inhibits ARHGAP26 silencing-induced ovarian cancer cell invasion and migration

**Fig. 5:**
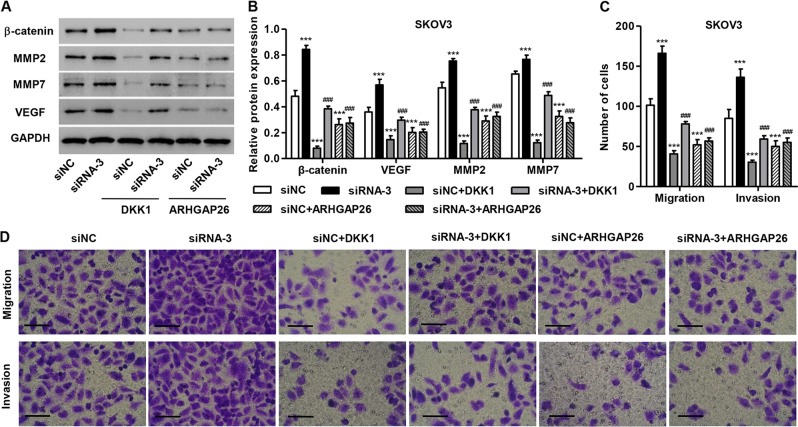
DKK1 treatment inhibited ARHGAP26 downregulation-induced invasion and migration of SKOV3 cells. SKOV3 cells transfected with AGRHAP26 siRNA-3 or siNC were treated with DKK1 (200 ng/mL) or transduced with recombined ARHGAP26 expression lentivirus, and expression of β-catenin, MMP2, MMP9, and VEGF (**a**, **b**), and SKOV3 cell migration and invasion (**c**, **d**) were detected via western blotting and Transwell analysis. Scale bars: 100 μm. ***P < 0.001 compared with siNC. ^###^*P* < 0.001 compared with siRNA-3

Next, we tried to explore the effects of β-catenin signaling on ARHGAP26-mediated ovarian tumorigenesis and an antagonist of the β-catenin pathway (DKK1) was introduced in SKOV3 cells. As expected, DKK1 treatment significantly reduced β-catenin expression in SKOV3 cells transfected with siNC or siRNA-3 (Fig. 5a, b). Importantly, DKK1 treatment significantly inhibited the migration and invasion of SKOV3 cells with silencing of ARHGAP26 (Fig. 5c, d). These results suggested that ARHGAP26 regulates ovarian cancer cell invasion and migration through β-catenin signaling.

### SMURF1 promotes ovarian cancer cell migration and invasion by inhibiting ARHGAP26

**Fig. 6:**
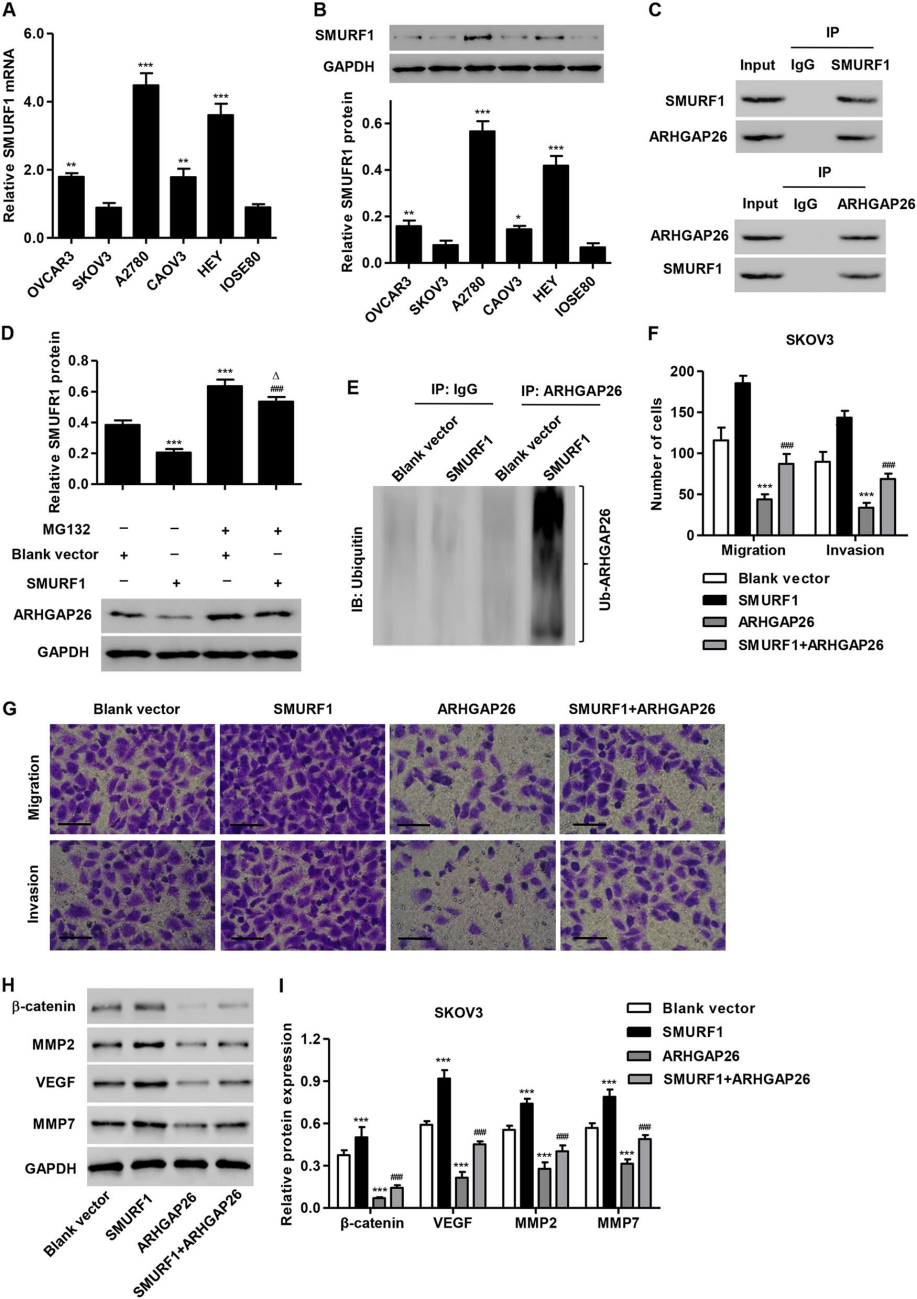
SMURF1 promotes ovarian cancer cell migration and invasion by inhibiting ARHGAP26. **a**, **b** SMURF1 expression in five ovarian cancer cell lines and the nonmalignant human ovarian surface epithelial cell line IOSE80 was detected. **c** SKOV3 cell lysates were subjected to immunoprecipitation with control IgG, or anti-SMURF1 or anti-ARHGAP26 antibody. The immunoprecipitates were then blotted with anti-SMURF1 or anti-ARHGAP26 antibody. **d** SKOV3 cells transduced with recombined pLVX-Puro-SMURF1 or control lentivirus (blank vector) were treated with MG132 (50 μM) for 4 h, and the expression of ARHGAP26 was determined by western blotting. **e** SKOV3 cells were transduced with recombined pLVX-Puro-SMURF1 or control lentivirus (blank vector). After 48 h, the cells were collected and AGRHAP26 was immunoprecipitated and immunoblotted with control IgG, or anti-ARHGAP26 or anti-ubiquitin antibody. SKOV3 cells were transduced with recombined pLVX-Puro-SMURF1, pLVX-Puro-ARHGAP26, or control lentivirus (blank vector), and cell migration, invasion (**f**, **g**), and expression of β-catenin, VEGF, MMP2, and MMP7 (**h**, **i**) were detected using Transwell and western blotting assays. Scale bars: 100 μm. *P < 0.05, **P < 0.01, and ***P < 0.001 compared with IOSE80 cells or cells transduced with blank vector. ^###^*P* < 0.001 compared with SMURF1. ^Δ^*P* < 0.05 compared with blank vector plus MG132

According to the UbiBrowser database (http://ubibrowser.ncpsb.org/), ARHGAP26 is predicted to be ubiquitinated by multiple E3 ubiquitin ligases, such as SMURF1, CBL, NEDD4, MDM2, and WWP1, which have been found to be dysregulated in ovarian cancer and associated with ovarian cancer cell proliferation and metastasis^15,19–22^, with a high and middle confidence interaction. Therefore, the associations between these E3 ubiquitin ligases and ARGHAP26 were further confirmed. We stably overexpressed SMURF1, CBL, NEDD4, MDM2, or WWP1 in SKOV3 cells and the expression of these proteins was significantly increased compared with that in control cells or cells treated with blank vector (Supplementary Fig. S2). SMURF1, CBL, NEDD4, or MDM2 overexpression significantly reduced the expression of ARHGAP26, with the lowest expression detected in SKOV3 cells with SMURF1 overexpression (Supplementary Fig. S3). Furthermore, SMURF1 expression levels in ovarian cancer cell lines were also measured. As shown in Fig. 6a, b, increased SMURF1 expression was observed in ovarian cancer cell lines compared with that in IOSE80 cells, with the lowest expression detected in SKOV3 cells. SMURF1 coimmunoprecipitated with ARHGAP26 (Fig. 6c). Reciprocal immunoprecipitation with ARHGAP26 antibodies also brought down SMURF1, suggesting an interaction between SMURF1 and ARHGAP26 in SKOV3 cells. In addition, the decrease in the ARHGAP26 level could be reversed by the addition of the proteasome inhibitor MG132, suggesting that SMURF1 regulates ARHGAP26 levels in a proteasome-dependent manner (Fig. 6d). Moreover, SMURF1 overexpression in SKOV3 cells significantly induced ubiquitination of ARHGAP26 (Fig. 6e). Importantly, SMURF1 overexpression significantly promoted the migration and invasion of SKOV3 cells (Fig. 6f, g), and increased the expression of β-catenin, VEGF, MMP2, and MMP7 (Fig. 6h, i), and these effects were reversed by ARHGAP26 overexpression.

### Expression of β-catenin and SMURF1, and correlation analyses in ovarian cancer tissues

**Fig. 7:**
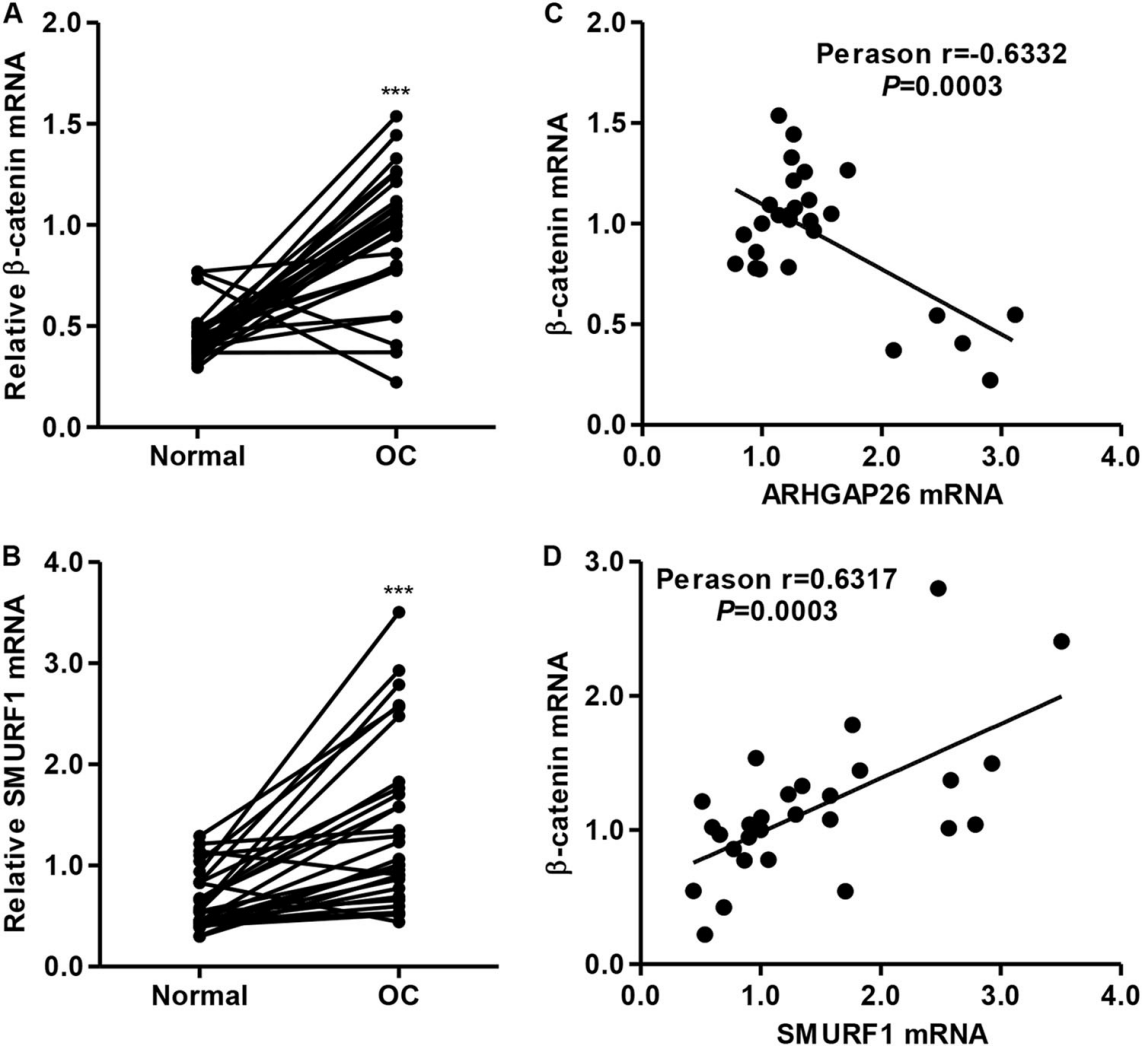
Expression of β-catenin and SMURF1 and correlation analysis in ovarian cancer tissues. **a**, **b** β-Catenin and SMURF1 mRNA expression in ovarian cancer tissues and adjacent normal ovarian tissues from our independent hospital database was detected using real-time PCR. **c**, **d** Pearson’s correlation scatter plots for ovarian cancer tissues (*n* = 28). ***P < 0.001 compared with normal. OC, ovarian cancer; normal, adjacent noncancerous tissues

We analyzed the mRNA expression levels of β-catenin and SMURF1 in ovarian cancer tissues and performed a Pearson’s correlation analysis between ARHGAP26 and β-catenin or SMURF1 and β-catenin. The results demonstrated upregulated mRNA expression of β-catenin and SMURF1 in ovarian cancer tissues compared with corresponding adjacent noncancerous ovarian tissues from our independent hospital cohort (Fig. 7a, b). ARHGAP26 mRNA expression was negatively correlated with the mRNA expression of β-catenin in ovarian cancer tissues (Fig. 7c), whereas SMURF1 mRNA expression was positively correlated with the mRNA expression of β-catenin in ovarian cancer tissues (Fig. 7d).

## Discussion

Ovarian cancer possesses great potential for growth and metastasis, resulting in higher morbidity, mortality, and health-care costs^23^. Herein, the present study assessed ARHGAP26 expression and its underlying mechanism in regulation of ovarian cancer cell proliferation, invasion, and migration. Our results provided evidence, suggesting that ARHGAP26 inhibits ovarian cancer cell invasion and migration in vivo and in vitro, and SMURF1-mediated ARHGAP26 ubiquitination may promote ovarian cancer cell invasion and migration via the β-catenin signaling pathway.

Previous studies showed significantly downregulated ARHGAP26 expression in glioblastoma and myeloid malignancies^9,11^. In the current study, we clarified for the first time that ARHGAP26 expression is decreased in ovarian cancer tissues compared with adjacent noncancerous ovarian tissues from TCGA and our independent hospital databases, and in ovarian cancer cell lines compared with nonmalignant human ovarian surface epithelial cells, and found that ovarian cancer patients with high ARHGAP26 expression tended to have a poor prognosis, suggesting that ARHGAP26 may act as a tumor suppressor involved in ovarian cancer development. ARHGAP26 disruption induced a dramatic loss of an epithelial cellular phenotype and increased gastric cancer cell invasion and migration^24^. Loss of ARHGAP26 activity also dramatically increased the invasion of SW480 colorectal cancer cells^25^. Here we demonstrated a similar role of ARHGAP26 in regulation of cell proliferation, invasion, and migration in ovarian cancer. ARHGAP26 overexpression inhibited ovarian cancer cell proliferation, migration, and invasion, and lung metastasis in xenograft nude mice bearing ovarian cancer, whereas ARHGAP26 silencing promoted these behaviors. Therefore, we came to the conclusion that ARHGAP26 is of great importance in the tumorigenesis of ovarian cancer.

Other remarkable findings of the present study were that ARHGAP26 overexpression inhibited the expression of β-catenin, VEGF, MMP2, and MMP7, whereas ARHGAP26 silencing promoted the expression of these proteins. These findings are consistent with GSEA data that shows negative regulation between ARHGAP26 and these proteins. β-catenin, the core of the classical Wnt signaling pathway, is highly expressed in many malignant tumors and associated with chemotherapy resistance, recurrence, and prognosis in ovarian cancer^26,27^. Moreover, β-catenin can increase the transcription of downstream β-catenin signaling pathway-related genes, including VEGF, MMP2, and MMP7, thereby leading to tumor cell proliferation and metastasis^28,29^. In the present study, ovarian cancer cells treated with DDK1, an antagonist of the β-catenin pathway, demonstrated decreased cell migration and invasion induced by ARHGAP26 silencing, indicating that ARHGAP26 regulates ovarian cancer cell invasion and migration by regulating VEGF, MMP2, and MMP7 expression through the β-catenin signaling pathway. ARHGAP26 is a GTPase-activating protein that was found to be inversely correlated with the expression of the GTP-bound form of RhoA (GTP-RhoA), the active form of RhoA. Moreover, a previous study reported that activated RhoA contributes to β-catenin accumulation, leading to an increase in cell migration^30^, which suggests that ARHGAP26 may regulate β-catenin expression through RhoA. However, inactivation of RhoA can contribute to colorectal cancer progression and metastasis through activation of Wnt/β-catenin signaling^31^. Therefore, the discrepancy between RhoA activity and β-catenin accumulation may be attributed to differences in cell types/tissues and it warrants further study. Moreover, β-catenin mRNA expression was found to be increased in ovarian cancer tissues and negatively correlated with ARHGAP26 mRNA expression.

Whether ARHGAP26 can be regulated in ovarian cancer remains an unanswered question. According to the UbiBrowser database (http://ubibrowser.ncpsb.org/), ARHGAP26 is predicted to be ubiquitinated by multiple E3 ubiquitin ligases, such as SMURF1, CBL, NEDD4, MDM2, and WWP1, which have been found to be dysregulated in ovarian cancer and associated with ovarian cancer cell proliferation and metastasis^15,19–22^. In this study, compared with other E3 ubiquitin ligases, the decrease in ARHGAP26 protein expression in cells with SMURF1 overexpression was the most significant and SMURF1 overexpression promoted ARHGAP26 ubiquitination and ovarian cancer cell invasion and migration, along with an increase in β-catenin, VEGF, MMP2, and MMP7 expression. Similarly, SMURF1 has been found to be increased in ovarian cancer patients and be associated with cell migration and invasion in ovarian cancer^15^. ARHGAP26 overexpression inhibited SMURF1-mediated cell migration, invasion, and β-catenin signaling in ovarian cancer, thereby suggesting that SMURF1-mediated ARHGAP26 ubiquitination may promote ovarian cancer cell invasion and migration via the β-catenin signaling pathway. Moreover, SMURF1 mRNA expression was positively correlated with β-catenin mRNA expression in ovarian cancer tissues.

In conclusion, the present study preliminarily provides insights into the anticancer effect of ARHGAP26 in ovarian cancer cells. ARHGAP26 overexpression inhibits the proliferation, migration, and invasion of ovarian cancer cells. SMURF1-mediated ubiquitination of ARHGAP26 may promote ovarian cancer cell invasion and migration through the β-catenin signaling pathway. Therefore, ARHGAP26 may be a promising novel therapeutic target for ovarian cancer treatment.

## Supplementary information

Supplementary Information
