# Supplementary material for: SMURF1-mediated ubiquitination of ARHGAP26 promotes ovarian cancer cell invasion and migration
Source: Exp Mol Med. 2019 Apr 19;51(4):46. doi: 10.1038/s12276-019-0236-0 (PMC6474862; doi:10.1038/s12276-019-0236-0)
Supplement: Supplementary file 1 — Supplementary Information [file 12276_2019_236_MOESM1_ESM.docx]

**SMURF1-mediated ubiquitination of ARHGAP26 promotes cell invasion and migration of ovarian cancer**

Xuri Chen^1^*, Shaoyun Chen^2^*, Yao Li^1^, Yanling Gao^1^, Shuying Huang^1^, Hongping Li^3^, Yuanfang Zhu^1,2^

^1^Department of Obstetrics and Gynecology, Bao’an Maternity and Child Health Hospital, Jinan University, Shenzhen 518100, China

^2^Maternal-Fetal Medicine Institute, Bao’an Maternity and Child Health Hospital, Jinan University, Shenzhen 518100, China

^3^Shenzhen children's Hospital, Shenzhen 518000, China

* Contributed equally

Corresponding author

Hongping Li, Shenzhen children's Hospital, No. 7019 Yitian Road, Fu'tian District, Shenzhen 518000, China. Tel.: +86-0755-83009810, Fax.: +86-0755-83009810, E-mail: [hongping_li@126.com](mailto:hongping_li@126.com)

Yuanfang Zhu, Department of Obstetrics and Gynecology, Bao’an Maternity and Child Health Hospital, Jinan University, Shenzhen 518100, China; Maternal-Fetal Medicine Institute, Bao’an Maternity and Child Health Hospital, Jinan University, No. 56 Yulv Road, Bao’an District, Shenzhen 518100, China. Tel.: +86-0755-27812637, Fax.: +86-0755-27812637, E-mail: [zhuzn0620@163.com](mailto:zhuzn0620@163.com)

**Running title:** SMURF1/ARHGAP26/β-catenin signaling in ovarian cancer

Total number of words: 3016


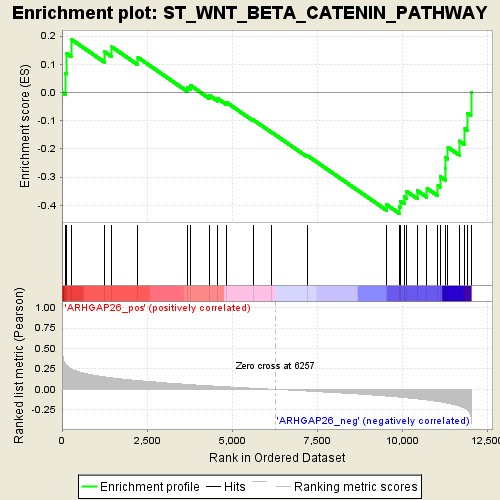


**Figure S1.** **Enrichment plots of GSEA.** GSEA demonstrated the genes of Wnt/β-catenin pathway were correlated with patients with ARHGAP26 low vs. ARHGAP26 high patients. *P*<0.001.


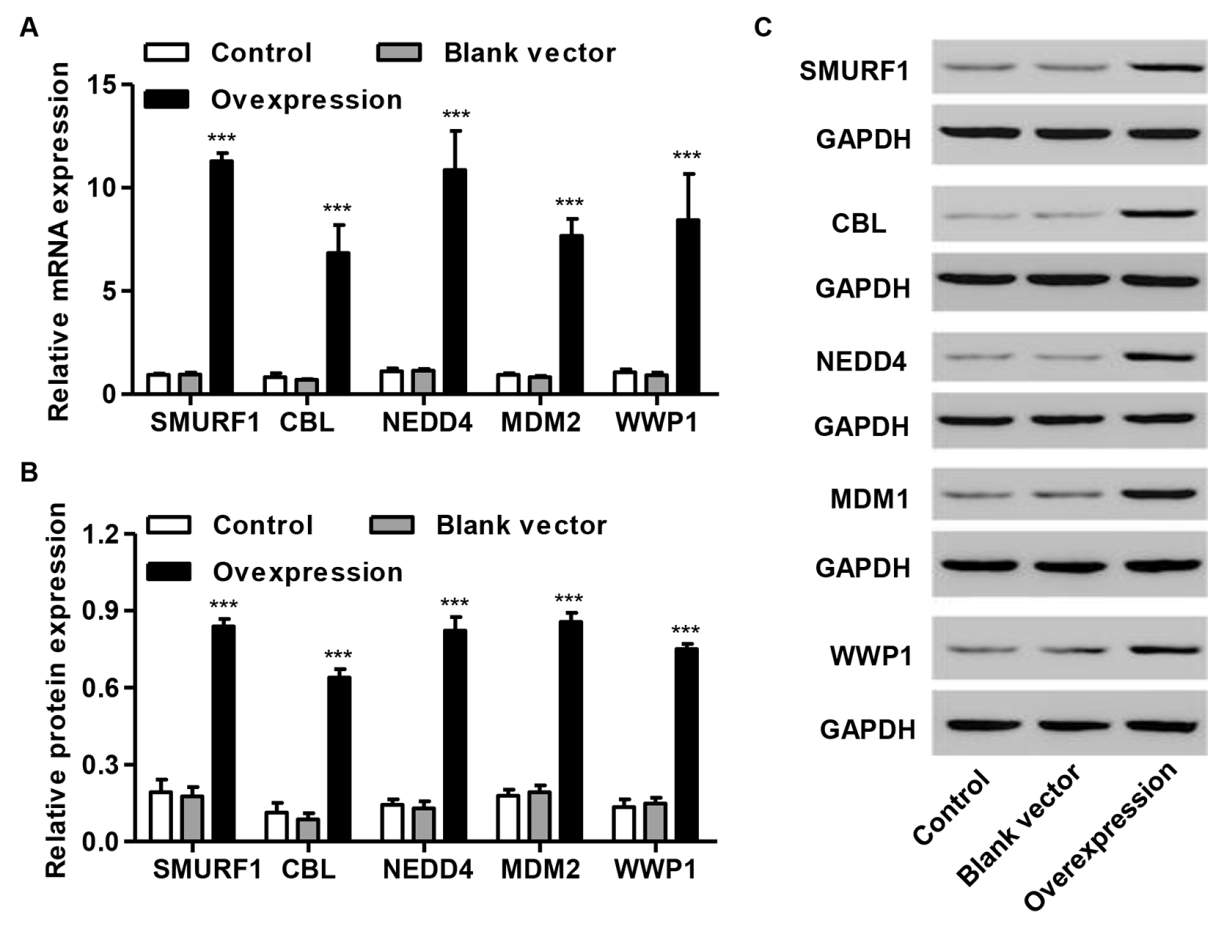


**Figure S2. SMURF1, CBL, NEDD4, MDM2, and WWP1 overexpression in SKOV3 cells.** SKOV3 cells were transduced with recombined pLVX-Puro-SMURF1, pLVX-Puro-CBL, pLVX-Puro-NEDD4, pLVX-Puro-MDM2, pLVX-Puro-WWP1 or control lentivirus (blank vector), and SMURF1, CBL, NEDD4, MDM2, and WWP1 expression was detected by Real-time PCR (A) and western blot (B, C). ****P*<0.001 compared with control.


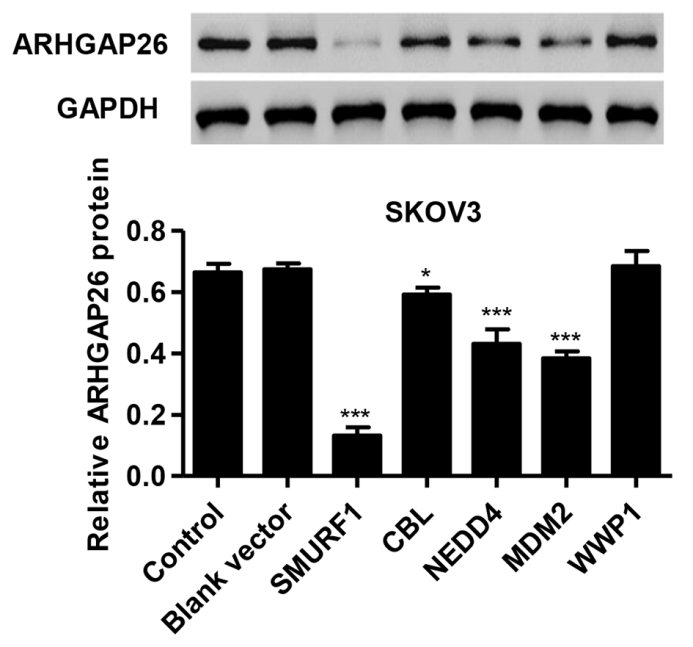


**Figure S3.** **ARHGAP26 protein expression in SKOV3 cells.** SKOV3 cells were transduced with recombined pLVX-Puro-SMURF1, pLVX-Puro-CBL, pLVX-Puro-NEDD4, pLVX-Puro-MDM2, pLVX-Puro-WWP1 or control lentivirus (blank vector), and AGRHAP26 expression was detected by western blot. **P*<0.05, ****P*<0.001 compared with control.
